# Injuries of Nerves

**Published:** 1903-12-05

**Authors:** 


### INJURIES OF NERVES.

In a clinical lecture delivered at the London Hospital Mr. James Sherren[1] discussed some points in connection with nerve injuries. He pointed out that conduction of impulses through nerves might be impaired or abolished by (1) anatomical division, the result of a wound or rupture of the nerve, (2) by contusion or by pressure applied for a longer or shorter period, or by (3) physiological division, meaning by the last that condition where conduction is entirely absent without there being any severance of the nerve sheath. There is no method by which a diagnosis can be made between anatomical and physiological division due to subcutaneous trauma short of cutting down on the nerve and seeing. This is unfortunate, for in the case of a nerve being completely severed immediate operation is called for.

In examining a case of suspected nerve injury it is well to follow out a definite plan, as follows:—(1) General inspection in which are noticed the position of the injured part or limb, the nature and site of wounds, scars, etc., and the condition of the skin, nails and hair. (2) Examination of muscles for wasting, paralysis and reaction to electrical stimulation. (3) Examination of sensation to light touch and to pin pricks. It is often possible to recognise that there has been some nerve injury by simply looking at the part, *e.g*, in the case of the musculo-spiral and ulnar nerves. By the wasting of muscles it may be seen that a certain group has been affected, and by testing the electrical reactions of the muscles the degree as well as the position of the nerve lesion may be estimated. In testing, the faradic and galvanic currents are used. The former is a stimulus to nerve fibres, the latter to muscle fibres. It is necessary in order to obtain good results, to use saline solution for moistening the electrodes and the skin, and the latter should be well soaked in the solution first. The faradic current is first applied; if the muscles react to this, it is clear that there is conduction through the nerve supplying the muscles that are being tested. If there is no contraction, the nerve is anatomically or physiologically divided. The galvanic current stimulates muscle and tells its condition. Muscle reacts to galvanism so long as there is any contractile substance left. It is important to notice two things when applying galvanic stimulation, firstly whether the character of the contraction is normal, and secondly whether reaction follows more readily when the negative pole is applied over the muscle than when the positive pole is used or *vice versâ*.

It is only when some time has elapsed since the injury that the electrical tests are reliable. The reactions are at first normal and faradic irritability is only lost about the fourth or fifth day after cutting off of the nerve supply.

In testing sensation it is necessary to use two methods, one for light touch and one for pin pricks. For the former the best way is to use a wisp of cotton wool and touch the skin lightly. In injuries of peripheral nerves the area of loss of sensation to pin pricks is never so extensive as the area for the loss of light touch. By marking out the area of loss for light touch it is possible to recognise what nerve is involved. The loss of sensation to pin pricks will tell the degree of the injury, whether there is partial or complete division of the nerve. If the loss for light touch and pricks together or separately is less than should be present in division, it shows that the nerve is not completely severed.

Mr. Sherren emphasises the importance of making it a rule to examine the nervous condition of the parts below wounds, as in the event of a nerve being divided primary suture offers a better prognosis than an operation undertaken after a considerable interval of time has elapsed.

[1] Clin. Jour., Nov. 11.

